# Creating a Career Development Path for Young Supply Chain Professionals: Three Case Studies in Benin, Kenya, and South Africa

**DOI:** 10.9745/GHSP-D-23-00320

**Published:** 2025-05-09

**Authors:** Rachel Msimuko, Ricardo Sedomedji Missihoun, Chloe Peebles, Jenny Froome, Lloyd Matowe, Pamela Steele

**Affiliations:** a Zambia Medicines and Medical Supplies Agency, Lusaka, Zambia.; b L’Association des Logisticiens Béninois, Cotonou, Benin.; c Global Health Supply Chain-Technical Assistance Francophone Task Order, Bogota, Colombia.; d Upavon, Midrand, South Africa.; e Partnership for Improving Supply Chain Management in Africa Limited, Lusaka, Zambia.; f Manica University, Lusaka, Zambia.; g Pamela Steele Associates, Kisumu, Kenya.

## Abstract

Low and middle-income countries (LMICs) continue to have a limited supply of skilled supply chain (SC) management professionals in the public sector. In addition, the SC workforce lacks the competencies demanded by new technologies and markets. Young people may be an untapped resource for the procurement and SC management workforce. We present 3 use cases in which career development paths were created for young SC professionals in Benin, Kenya, and South Africa. In Benin, with advocacy from L’Association des Logisticiens Béninois, the professional body of logisticians, career development opportunities were made through creating specific programs like the Young Logisticians Professionals Program. SAPICS, the professional body for SC management in South Africa, has been providing opportunities for career development by giving students and young professionals access to subject matter experts, conferences, site visits, and various industry-specific training and networking opportunities, as well as general “job readiness” training through coaching and mentorship programs. In Kenya, the Girls on the Move program focuses on introducing SC management as a career path to girls through internships, skills training, and mentorship, all aimed at equipping them for successful work placements. The COVID-19 pandemic underscored the critical importance of SC management and highlighted vulnerabilities that demand greater responsiveness and resilience. In the post-COVID era, it is imperative for the public SC sector to build an agile and skilled workforce capable of addressing immediate needs and supporting long-term pandemic preparedness. The cases presented show how opportunities for training, mentorship, and work experience were made available to young professionals and highlight some of the positive outcomes of these initiatives. The cases also illustrate innovative approaches to developing career pathways for youth in LMICs that aim to expand the pool of skilled professionals who can strengthen public SCs and enhance their capacity to respond to future challenges.

## Abstract (precis)

Creating career development paths for young supply chain professionals in LMICs provides the youth with needed employment and entrepreneurship opportunities. This can help to unlock their potential and contribute to better supply chain performance in the public health sector.

## Key Messages

- There is a limited supply of skilled supply chain professionals to address the longstanding and emerging challenges in the public health sector supply chain.
- Support from professional bodies can contribute to creating an enabling environment for youth employment and entrepreneurship and accelerate the professionalization of the supply chain workforce.
- Creating career development paths for young supply chain professionals provides them with employment and entrepreneurship opportunities and may address the need for a skilled workforce in the supply chain sector.
- We present cases from Benin, Kenya, and South Africa that demonstrate that investing in skills training and creating supportive environments for young supply chain professionals to enter the industry contributes to better supply chain outcomes.

## INTRODUCTION

The performance of supply chains in low- and middle-income countries (LMICs) has a direct impact on the availability of health commodities and, consequently, the quality of health service delivery. A baseline assessment of the World Health Organization’s target for the availability and affordability of essential medicines to treat noncommunicable diseases, conducted between 2008 and 2015, found that in low-income countries, the median generic availability across all medicines was 40.2% in the public sector and 59.1% in the private sector.^1^ This is significantly below the World Health Organization target of 80% availability by 2025. Poor supply chain performance is one of the factors contributing to these shortfalls, hindering progress toward achieving universal health coverage, with 3.1 billion people globally lacking effective universal health coverage as of 2023.^2^

A major challenge impacting supply chain performance is the limited supply of skilled supply chain management labor. Current workforces often lack the competencies required by new technologies and markets to deliver health commodities in the right quantities and at the right time. For example, the absence of technical expertise in advanced supply chain technologies limits the potential of these tools to improve visibility and decision-making. Furthermore, the lack of skilled supply chain professionals to forecast demand and manage complex procurement processes often results in delayed deliveries and stock-outs, particularly during periods of rapid demand shifts, such as those seen during pandemics.

The talent deficit in the public-sector medicine supply chain is significant at all levels of the system. Attracting and retaining supply chain technical talent and leadership has been identified as a key factor in improving supply chain performance.^3^ Public-sector supply chain management is also inherently complex, comprising various organizations and tasks.^4^ Comparisons with the private sector reveal that competencies in the public sector tend to be lower.^5^ Addressing these issues requires governments and stakeholders to consider both supply factors—such as training and talent availability—and demand factors, including ensuring sufficient funding, defining appropriate competencies, and creating roles that address workforce gaps.

However, there are few formal skills development programs for those entering the supply chain sector. This gap is further compounded by a lack of staffing structures in the health sector that are specifically designed for supply chain professionals. The absence of clear career development pathways discourages youth from pursuing careers in supply chain management, representing a missed opportunity to attract capable professionals who could contribute to improved supply chain performance. Additionally, misunderstandings about supply chain management and the roles it offers exacerbate the problem, leaving potential talent untapped. High levels of youth unemployment, which according to International Labour Organization stood at 12.56% for ages 15 to 24 years in 2024,^6^ also remain a problem. Initiatives that develop the skills needed to enter the supply chain profession may help in responding to the human resource needs in the supply chain sector and may also provide more opportunities for youth employment. Engaging youth in the supply chain introduces new talent and perspectives into the supply chain workforce that may contribute to better systems-strengthening initiatives.

There are few formal skills-development programs for those entering the supply chain sector.

In this article, we present use cases drawn from Benin, Kenya, and South Africa to show ways in which young supply chain professionals have been supported to develop the skills needed for the public supply chain sector.

## YOUNG LOGISTICIANS PROFESSIONALS PROGRAM IN BENIN

The results of a 2015 assessment of the Beninese supply chain showed a lack of skilled human resources and poor reporting, making it difficult for health commodities to be continuously available at pharmacies and hospitals, particularly in remote areas of Benin. To tackle this problem, L’Association des Logisticiens Béninois (ASLOB) (Association of Beninese Logisticians), a nongovernmental organization founded in Benin in 2007, worked to create opportunities for young professionals in the supply chain field. This effort was driven by the recognition of the country’s supply chain challenges and the potential of youth to address them.

To improve access to essential products through effective supply chains managed by skilled professionals,^7^ ASLOB implemented various initiatives, particularly in the health sector. One key focus was building partnerships with organizations, such as the Platforme du Secteur Sanitaire Privé, as well as donors, the Ministry of Health (MOH), the regional public health institute, academia, and the International Association of Public Health Logisticians. Through these partnerships, ASLOB collaborated with academic institutions, like the École Nationale d’Economie Appliquée et de Management (National School of Applied Economics and Management), to support students interested in public health logistics and transport programs. Additionally, ASLOB established a network of youth supply chain professionals and researchers connected to schools, providing services for their professional development,^7^ growth, employment, and entrepreneurship.

ASLOB worked with sister country associations to initiate the establishment of the West Africa Logisticians Associations Federation and the African Logisticians Associations Federation to expand the role of national supply chain professional associations and regional federations in supporting the growth, employment, and entrepreneurship of young professionals across Africa.^8^

To create opportunities for career development, ASLOB provided training, capacity-building, internships, and consultancy opportunities for young professionals in logistics and data analytics, enabling them to contribute to development projects and research. ASLOB also advocated the professionalization of health supply chain management at the national level through its membership in the coalition representing Francophone countries’ supply chain professional associations.

ASLOB facilitated partnerships between Benin’s Platforme du Secteur Sanitaire Privé, the Global Health Supply Chain-Technical Assistance (GHSC-TA) Francophone Task Order (TO) project, and other stakeholders to support and implement the Young Logisticians Professionals Program (YLPP) in 2018. The program engaged young professionals, tapping into an underused demographic to build a skilled labor pool capable of improving supply chain operations, data reporting accuracy, and stock management. By integrating youth into the workforce, the program also addressed the broader issue of youth unemployment, fostering professional growth and contributing to economic development. The MOH provided oversight for the program and ensured integration into national systems, and the GHSC-TA Francophone TO project supported training, evaluation, and implementation of the program.

The YLPP tapped into an underused demographic to build a skilled labor pool capable of improving supply chain operations, data reporting accuracy, and stock management.

In December 2019, the GHSC-TA Francophone TO project, working closely with the MOH, evaluated the program’s impact on the stock status of 75 key tracer health commodities—such as antimalarial and family planning items—across 104 health centers in 26 health zones in Benin. The evaluation compared survey data collected over a 6-month implementation period from both YLPP-supported and non-YLPP-supported sites.

The survey results showed that for young adult malaria medication, the YLPP-supported facilities had lower stock-out rates (4.48% vs. 24.24%), understock rates (2.28% vs. 12.28%), and overstock rates (23.88% vs. 33.33%), compared to non-YLPP-supported facilities.^9^ The survey results also showed increased stock card accuracy and improved stock management and reporting rates (97% and 48%, respectively). YLPP-supported facilities were nearly twice as likely to have sufficient stock of antimalarial treatments (73% versus 49%) and family planning commodities (56% versus 25% for cycle beads and 56.25% versus 25% for male condoms, respectively).

In health facilities benefiting from YLPP support, sites had staff with the requisite skills to manage inventory and produce timely reports to trigger replenishments. The evaluation results showed how the approach had resulted in logistical data improvements.

The program’s success led to its expansion through the World Bank-funded Sahel Women’s Empowerment and Demographics Project, which extended YLPP to 48 additional communes and added 77 participants. The Beninese government also supported the program’s growth, incorporating 34 additional participants in health zone warehouses. The YLPP serves as a replicable model for addressing supply chain challenges through youth engagement. By fostering partnerships, offering targeted training, and using evidence-based advocacy, the YLPP demonstrated how investing in local talent can create a sustainable workforce, improve supply chain performance, and enhance health outcomes. This approach provides valuable lessons for other regions and countries seeking to address similar challenges in their supply chains.

## GIRLS ON THE MOVE PROGRAM IN KENYA

Despite its critical role in economic and organizational efficiency, the supply chain sector has been marked by a significant need for more skilled female labor. Women bring a unique set of skills, perspectives, and knowledge that are often missing in health care management and decision-making, particularly in the procurement and distribution of health products. However, women remain underrepresented in the global supply chain sector, comprising only 43% of the workforce and just 27% of management positions.^10^ Recognizing the transformative potential of increasing gender diversity in supply chains, the Girls on the Move program in Kenya was designed to address high unemployment rates among girls and young women while tackling gender disparities in the supply chain sector.

As a pilot initiative, the program, launched in 2022 by Pamela Steele Associates (PSA) with seed funding from the Reproductive Health Services Coalition, engaged youth by targeting unemployed female supply chain graduates aged 18–29 years and introducing them to career opportunities in the supply chain sector. A total of 36 participants were enrolled in an internship program that combined 8 months of work placements with structured skills training and mentorship. Participants were strategically matched with public and private sector organizations, including health facilities, hospitality businesses, and government agencies in Kisumu and Nairobi County in Kenya. These partnerships provided interns with practical experience, supported by PSA’s oversight, bimonthly training workshops, and mentorship sessions.

The program produced significant positive outcomes. By the end of the internship period, 15 participants had secured employment by March 2023, which increased to 21 by November 2024. Interns reported improved confidence and skills in inventory management, procurement, and warehouse operations. Employers also observed the positive effects of workplace diversity. One participating organization was motivated to establish its first supply chain management department, highlighting the program’s potential to drive systemic change.

Participants and employers provided positive feedback about the program’s effectiveness. Monthly learning logs, bimonthly workshops, and pre- and post-program assessments gathered valuable insights. Interns valued the hands-on experience and mentorship, which helped connect their academic knowledge with real-world professional skills. Employers expressed high satisfaction, with many requesting more interns and supporting the expansion of the program.

Encouraged by the success of its pilot program, VillageReach approached PSA to initiate plans for scaling up the Girls on the Move program beyond Kisumu and Nairobi to additional counties and countries to train 500 graduates over the next 5 years. The pilot phase underscored the significance of solid local partnerships, customized training, and mentorship in achieving impactful outcomes. These elements have been crucial in addressing youth unemployment and the skills gap in the supply chain, demonstrating the transformative potential of targeted, gender-focused initiatives in workforce development.

## THE STUDENT/YOUNG PROFESSIONAL DEVELOPMENT INITIATIVE IN SOUTH AFRICA

Work readiness is an issue of interest for most employers in recruiting university graduates in all fields. Some graduates’ on-the-job performance has been discovered to be below the standards expected for their entry-level positions, leading to unmet employer expectations.^11^ This challenge is particularly pronounced in supply chain management, where operational inefficiencies could have dire consequences, such as delays in medicine availability in the health sector. SAPICS, the professional body for supply chain management in South Africa, recognized that students leaving university and higher education institutions were not fully prepared to meet industry expectations despite having sound technical knowledge. The SAPICS Student/Young Professional Development Program Initiative was developed to bridge the gap between academic knowledge and industry expectations in supply chain management.

The Student/Young Professional Development Program Initiative was developed to bridge the gap between academic knowledge and industry expectations in supply chain management.

This program is targeted to a broad audience of students and young supply chain professionals across industries and is not limited to the public health sector. Participants were provided with memberships in a community of supply chain professionals and gained access to a range of skills and career development opportunities. These included sponsorships to attend industry events, networking with peers and experienced professionals, technical site visits to factories, warehouses, and distribution centers, and mentorship programs. Training modules focused on essential skills such as corporate protocols, personal branding, business communication, and career planning.

A significant component of this initiative is the Annual Young Professional Conference, hosted by SAPICS since 2014, which offers practical insights into supply chain management. The conference features diverse topics, ranging from technical supply chain processes to soft skills necessary for personal and professional development. The conference has consistently attracted a significant number of participants, including students, young professionals, and industry experts, indicative of a robust engagement from the supply chain community, reflecting the event’s importance in fostering industry readiness among emerging professionals.

During the COVID-19 pandemic lockdown in 2020, SAPICS quickly adapted by shifting to online sessions and virtual mentorship opportunities, ensuring continued support for students and young professionals during a challenging time.

In 2021, SAPICS took a significant step toward sustainability by forming a youth-led committee to design and manage the annual conference and related events. This approach empowered young professionals to take ownership of their development and allowed the program to evolve and grow over time.

SAPICS has reinforced its commitment to developing South African youth by rolling out new initiatives and partnerships aimed at upskilling and empowering young graduates and professionals for career success, addressing the skills gap in the supply chain field by establishing supply chain chapters or desks at selected universities giving students access to information on vacation work opportunities. With the University of South Africa, SAPICS also launched a series of classes to address the soft skills that supply chain graduates lacked.^12^

The success of this initiative highlights its broader potential as a model for professional associations across Africa and beyond. By fostering partnerships with academic and industry stakeholders, the program demonstrated how targeted training and mentorship could address workforce readiness challenges while creating pathways for young professionals. SAPICS’s initiative serves as a replicable framework for others seeking to enhance work readiness in supply chain management and other critical sectors. Through the lessons learned, professional organizations can build similar programs to bridge the gap between academia and industry, ensuring a pipeline of skilled and capable professionals ready to meet the demands of the modern workforce.

## DISCUSSION

As the world faces ongoing supply chain challenges, particularly in the health sector, there is an urgent need for quick decision-making and swift implementation of interventions to enhance the growth and resilience of supply chains. Central to these efforts is the recognition that supply chains rely on a skilled workforce capable of addressing and adapting to emerging global challenges. The use cases highlight initiatives by professional bodies, private firms, donors, development partners, and governments that focus on providing skills training to youth to strengthen the supply chain management workforce. These cases demonstrate that investing in skills training and creating supportive environments for young supply chain professionals to enter the industry contributed to getting better supply chain outcomes. It is hoped that lessons learned from the implementation of such programs may be used in the initiation of similar programs aimed at creating career pathways for the supply chain workforce that will improve the skills and capacity available to manage public health supply chains.

Initiatives that attract and support young supply chain professionals, offering them viable careers, are expected to play a role in improving access to medicines. This, in turn, can contribute to achieving universal health coverage and the health-related Sustainable Development Goals.

Establishing career development paths for young supply chain professionals is an important step in addressing long-standing and emerging challenges in health supply chains. However, while youth-focused initiatives provide a crucial pipeline of skilled professionals, they must be integrated into a broader public sector supply chain management health workforce strategy to achieve sustainable improvements. A youth-focused approach can serve as a complementary component of a comprehensive plan that addresses both supply and demand aspects of the workforce.

Youth-focused programs contribute to this larger framework by injecting new talent and fresh perspectives into the workforce, but they must be supported by strategies that retain talent, promote career progression, and address gaps across all age groups and levels of expertise.

## CONCLUSION

In conclusion, while youth engagement in supply chain management is a valuable step toward enhancing workforce capacity, it is only one piece of the puzzle. A systematic solution that integrates youth-focused initiatives into a wider public sector supply chain management health workforce strategy, addressing both supply and demand factors, is essential. We believe that such a strategy may not only strengthen health supply chains but also contribute to ensuring their long-term sustainability and adaptability to future challenges. By planning for, establishing, and maintaining a robust workforce, the health sector can improve supply chain performance and contribute to better health outcomes globally.

## Notes

**Peer Reviewed**

**First Published Online:** January 14, 2025.
